# Alzheimer’s Disease-Associated SNP rs708727 in *SLC41A1* May Increase Risk for Parkinson’s Disease: Report from Enlarged Slovak Study

**DOI:** 10.3390/ijms23031604

**Published:** 2022-01-29

**Authors:** Michal Cibulka, Maria Brodnanova, Marian Grendar, Jan Necpal, Jan Benetin, Vladimir Han, Egon Kurca, Vladimir Nosal, Matej Skorvanek, Branislav Vesely, Andrea Stanclova, Zora Lasabova, Zuzana Pös, Tomas Szemes, Stanislav Stuchlik, Milan Grofik, Martin Kolisek

**Affiliations:** 1 Biomedical Centre Martin, Jessenius Faculty of Medicine in Martin, Comenius University in Bratislava, 03601 Martin, Slovakia; michal.cibulka@uniba.sk (M.C.); maria.brodnanova@uniba.sk (M.B.); marian.grendar@uniba.sk (M.G.); 2 Clinic of Neurology, AGEL Hospital in Zvolen, 96001 Zvolen, Slovakia; necpal.neuro@gmail.com; 3 Clinic of Neurology, University Hospital Bratislava, Slovak Medical University in Bratislava, 83303 Bratislva, Slovakia; benetinj@gmail.com; 4 Clinic of Neurology, University Hospital of L. Pasteur in Kosice, University of Pavol Jozef Safarik, 04066 Kosice, Slovakia; vladimir.han@gmail.com (V.H.); mskorvanek@gmail.com (M.S.); 5 Clinic of Neurology, University Hospital Martin, Jessenius Faculty of Medicine in Martin, Comenius University in Bratislava, 03601 Martin, Slovakia; egonkurca@gmail.com (E.K.); vladimir.nosal@uniba.sk (V.N.); 6 Clinic of Neurology, Faculty Hospital in Nitra, Constantine the Philosopher University in Nitra, 94901 Nitra, Slovakia; brano.vesely@yahoo.com; 7 Institute of Molecular Biology and Genomics, Jessenius Faculty of Medicine in Martin, Comenius University in Bratislava, 03601 Martin, Slovakia; avoj2017@gmail.com (A.S.); zora.lasabova@uniba.sk (Z.L.); 8 Department of Molecular Biology, Faculty of Natural Sciences, Comenius University in Bratislava, 84104 Bratislava, Slovakia; kubiritova.zuzka@gmail.com (Z.P.); tomas.szemes@uniba.sk (T.S.); stanislav.stuchlik@uniba.sk (S.S.); 9 GENETON s.r.o., 84104 Bratislava, Slovakia

**Keywords:** Parkinson’s disease, Alzheimer’s disease, *PARK16*, Na^+^/Mg^2+^ exchanger, *SLC41A1*, single nucleotide polymorphism

## Abstract

*SLC41A1* (*A1*) SNPs rs11240569 and rs823156 are associated with altered risk for Parkinson’s disease (PD), predominantly in Asian populations, and rs708727 has been linked to Alzheimer’s disease (AD). In this study, we have examined a potential association of the three aforementioned SNPs and of rs9438393, rs56152218, and rs61822602 (all three lying in the *A1* promoter region) with PD in the Slovak population. Out of the six tested SNPs, we have identified only rs708727 as being associated with an increased risk for PD onset in Slovaks. The minor allele (A) in rs708727 is associated with PD in dominant and completely over-dominant genetic models (OR_D_ = 1.36 (1.05–1.77), *p* = 0.02, and OR_COD_ = 1.34 (1.04–1.72), *p* = 0.02). Furthermore, the genotypic triplet GG_(rs708727)_ + AG_(rs823156)_ + CC_(rs61822602)_ might be clinically relevant despite showing a medium (*h* ≥ 0.5) size difference (*h* = 0.522) between the PD and the control populations. RandomForest modeling has identified the power of the tested SNPs for discriminating between PD-patients and the controls to be essentially zero. The identified association of rs708727 with PD in the Slovak population leads us to hypothesize that this *A1* polymorphism, which is involved in the epigenetic regulation of the expression of the AD-linked gene *PM20D1*, is also involved in the pathoetiology of PD (or universally in neurodegeneration) through the same or similar mechanism as in AD.

## 1. Introduction

The role of magnesium (Mg) homeostasis (MgH) in the pathoetiology of Parkinson’s disease (PD) is the subject of ongoing research and debate. The broad range of Mg actions in cellular physiology and at the level of the organism substantiates the assumption that disturbed MgH contributes to the degenerative processes associated with PD.

Magnesium is essential for cellular energetics [1,2]. It is required for ATP production, the stabilization of its structure, and its biological activity [1,2,3]. Overall, it interferes with mitochondrial homeostasis (MH) at various levels, from the structural organization of the mitochondria to various processes of mitochondrial respiration [1,2,3,4,5,6]. The close relationship between MgH and MH is also illustrated by the mitochondria serving as the main reservoir of Mg^2+^ in the cell [7]. Furthermore, Mg has anti-apoptotic, pro-proliferative, and pro-growth properties, and constituents of Mg homeostatic machinery interfere with cellular Akt/PKB and Erk1/2 pro-survival signaling [8,9,10].

Neurodegenerative diseases, including PD, are characterized at the cellular level by damage to MH and to the energy stability of the cell resulting primarily from aberrant mitophagy, ER-stress management, retromer function, ubiquitination, and adjacent protein turnover, i.e., processes that are tightly connected under normal physiological conditions [11,12,13]. The role of Mg in these processes is only marginally understood. Nevertheless, both the cytoplasmic pool (secured primarily by the chanzyme TRPM7) and the intramitochondrial/matrix pool (secured by the superconductive Mg^2+^ ion channel Mrs2) of Mg^2+^ are known to be particularly important for the maintenance of the membrane potential on the inner mitochondrial membrane (Δψ_m_) [2,4,5,6]. Any drop of matrix [Mg^2+^} attributable to the Mg-starvation of the cells, or to the dysfunction of Mrs2, induces a depolarization that further triggers mitophagy [14]. Recently, Zhao and colleagues have demonstrated that high glucose induces a drop of intracellular [Mg^2+^] accompanied by the induction of mitophagy in hFOB1.19 cells (conditionally immortalized fetal osteoblasts, ATCC CRL-11372^™^) [15].

The cytoplasmic and, indirectly, the intra-organelle (mitochondrial, ER, Golgi) concentrations of Mg^2+^ are dependent on the Mg^2+^ transporters, which constitute the Mg^2+^ transport circuit of the cytoplasmic membrane [6]. These transporters are as follows: (1) the chanzyme TRPM7, the major cellular Mg^2+^ influx gateway, and (2) the Na^+^/Mg^2+^ exchanger (NME) SLC41A1 (further referred as to A1), the major cellular Mg^2+^ efflux gateway [6,16,17]. Indeed, Cornell’s group has hypothesized that the published epidemiological data “support the possibility that mutations in genes relevant to MgH would alter PD risk” but warrant “deeper genetic analyses of PD patients” for confirmation that *SLC41A1* (further referred as to *A1*) and *TRPM7* are among these genes [18].

With regard to the possible involvement of NME A1 in the onset and progression of PD, Tucci and colleagues have identified two novel coding variants of genes that are from the *PARK16* locus and that are present only in the PD cohort, namely *RAB7L1* (c.470A > G; p.K157R) and *A1* (c.1049C > T, p.A350V) [19]. The former group of Kolisek has characterized *A1* c.1049C > T as a gain of function mutation resulting in “enhanced Mg^2+^-efflux conducted by A1 variant p.A350V”, which might lead, in the long-term, to “chronic intracellular Mg^2+^-deficiency, a condition that is found in various brain regions of PD patients and that exacerbates processes triggering neuronal damage” [20]. Lin et al. have subsequently identified a rare loss of function variant of A1 p.R244H in a cohort of 80 patients diagnosed with early onset of PD [21]. The mechanism behind the loss of function of A1 is a matter of speculation, although, Tatarkova and colleagues have recently provided data making it clear that “the presence or absence, and thus the functionality, of A1 influences mitochondrial processes involved in energy production” [1]. Moreover, Li and colleagues have recently associated the A1 variant p.R285Q with PD [22]. Further experimental evidence of the possible involvement of A1 in PD has been provided by Lin and coworkers who have shown that Mg sulfate (MgSO_4_) possibly protects SH-SY5Y cells against the neurotoxicity of 6-hydroxydopamine (6-OHDA) [23]. They have additionally demonstrated that 6-OHDA decreases the expression of *A1* (and other magnesiotropic genes) in 6-OHDA-treated SH-SY5Y cells, and that MgSO_4_ can reverse its decline [23]. The same group has also provided data revealing that, in a rat PD model, 6-OHDA alters the expression of *A1*/A1 (at both the RNA and protein levels), and that the extent of this alteration is responsive to [MgSO_4_] [23].

The *PARK16* locus comprises five genes, namely *SLC45A3*, *NUCKS1*, *RAB7L1*, *A1*, and *PM20D1* [24]. Its role in the susceptibility to PD has been pointed out by numerous genome-wide association studies (GWAS) and case-control studies. Three *A1* single nucleotide polymorphisms (SNP(s)) have been extensively studied with respect to their association with PD.

The major G allele of the *A1* polymorphism rs11240569 (for characteristics see Table 1) of a Han cohort in China has been shown to reduce the risk of idiopathic PD, with people who have the GG and AG genotypes exhibiting a reduced risk compared with those who have the AA genotype [25]. A similar outcome has been obtained in a study performed with an Iranian cohort [26].

Another *A1* SNP, rs708727 (Table 1), has been studied in a UK cohort, but no association between this SNP and PD has been found [19]. However, this SNP has been linked to Alzheimer’s disease (AD) [27].

In relation to PD, rs823156 (Table 1), is probably the most intensely studied, but is also the most controversial among the *A1* SNPs. This SNP has been associated with PD in cohorts from mainland China [28], Japan [29], and Korea [30], but not in cohorts from Eastern China [31], the north of Spain [32], and Malaysia [33]. Bai and colleagues have predicted, following in silico analyses, that rs823156 as a noncoding variant of *A1* “might affect PD risk by altering the transcription factor-binding capability of the genes” [34].

Previously published work has made it obvious that cells regulate the extent of Mg^2+^ efflux via A1 at the level of proteins and at the level of transcription [17,20,35,36]. However, the amount of information about the organization of the promoter of *A1* and its transcription-binding capacity is rather scarce [34].

In 2019, we published a study showing that the three aforementioned *A1* SNPs are not associated with any susceptibility toward PD in the Slovak population, as demonstrated by the means of frequentist statistics and by machine learning [37]. A major limitation of that study might have been the relatively low number of participants in both the PD (150) and the control (120) cohorts. Therefore, the aim of this study has been twofold, as follows: (1) to elucidate any possible association of rs11240569, rs708727, and rs823156 in a larger group of PD patients (150 + 358) and control probands (120 + 352), and (2) to sequence the promoter region of *A1* in a sub-cohort of PD samples in order to identify any possible SNPs within the promoter region and to examine their possible association with PD.

## 2. Results

### 2.1. Sequencing of SLC41A1 Promoter Region

**Figure 1:**
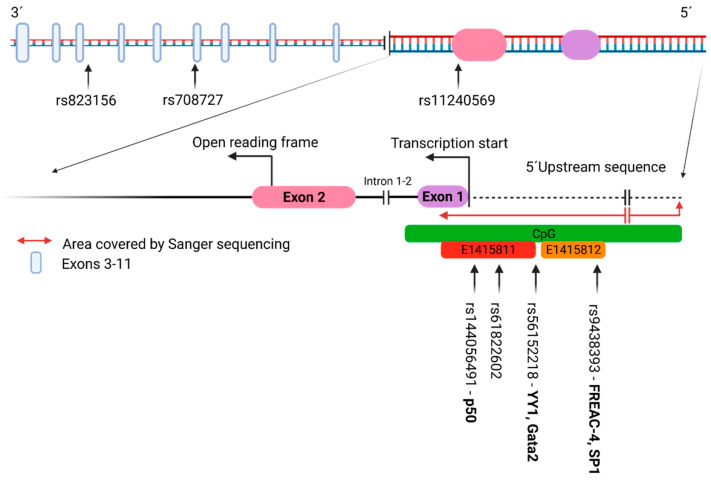
Gene organization of *A1* including adjacent upstream 5′UTR. According to Ensembl Transcript: *SLC41A1*-201 ENST00000367137.4, this gene is located on chromosome 1 and consists of 11 exons. Exon 1 represents 5′UTR (untranslated region), and exon 2 contains a part of this 5′UTR. 3′UTR is included in exon 11. In our previous study, we studied three SNPs (single nucleotide variants), namely rs11240569, rs708727, and rs823156 in *A1* [37]. In this work, we analyzed a sequence (1638 bp in length) located upstream of this gene. This sequence covers the 5′upstream sequence and, partially, exon 1. According to the UCSC genome browser [39], the sequence is a regulatory region represented by CpG islands (green rectangle). A promoter-like signature (EH38E1415811) and a proximal enhancer-like signature (EH38E14112) (red and orange rectangle, respectively) have been described in this region. We have identified four SNPs (rs144056491, rs61822602, rs56152218, and rs9438393) in this sequence. At rs144056491, a search within the reference sequence and then in the sequence with the variant resulted in the identification of a binding site for transcription factor p50. At rs9438393, the search resulted in the identification of a binding site for transcription factor FREAC-4 (the A allele). However, no binding site was detected in the variant sequence (G allele). At the same SNP, the G allele allows the binding of SP1. At rs56152218, the dominant T allele enables the binding of Gata2, and the minor allele that of YY1.

The Sanger sequencing and sequences analysis was performed in a sub-cohort of 96 PD patients (all from the PD Center in Martin). A fragment of the *A1* promoter region was studied, spanning from position 205,814,626 to 205,812,988 on chromosome one. The sequence of the fragment was chosen according to the Genecopoeia database [www.genecopoeia.com/product/search/view_seq_promoter.php?cid=&type=promoter&prod_id=HPRM53412 (accessed on 2 May 2018)]. The gene organization of *A1* and of its promoter/regulatory sequences is depicted in Figure 1. The sequencing allowed the identification of the following four SNPs in the *A1* promoter region: rs9438393, rs56152218, rs61822602, and rs144056491 (Figure 1). Next, we utilized the RFLP strategy to examine rs9438393 (restriction with *Hpy*166II), rs56152218 (restriction with *NIa*III), and rs61822602 (restriction with *Bmr*I) in a sub-group of 100 control samples. The SNP rs144056491 was not examined in the control group because of the lack of a suitable restriction enzyme.

ConSite [38] (Table 2), a web-based tool for finding cis-regulatory elements in genomic sequences, was employed to examine whether the variant (minor) allele for each of the four aforementioned SNPs altered the TF-binding profile of the *A1* promoter by rendering a new TF-binding site or by erasing the existing one. As input, we used 33 bp long sequences, one with the reference (major) allele and other with the variant (minor) allele for each SNP, respectively. At rs144056491, which is located within the binding site of transcription factor p50, both the major allele (C) and the minor allele (-, CC, CCC) presumably allow the binding of this transcription factor (Figure 1). The presence of the major allele (A) at rs9438393 might permit the binding of the transcription factor FREAC-4 (Table 2, Figure 1). However, if the minor allele (G) is present, then the FREAC-4 binding site is no longer recognized by the TF-binding predictive software. At the same SNP, the minor allele putatively allows the binding of SP1, which is not the case in the presence of the major allele (Table 2, Figure 1). The major allele (T) at rs56152218 putatively allows the binding of Gata2, but according to the prediction, this will not be the case in the presence of the minor allele (C). On the other hand, YY1 might bind the minor C-allelic variant, but not the major T-allelic variant (Table 2, Figure 1). According to the in silico prediction, SNP rs61822602 is not located within any TF-binding sequences (Table 2, Figure 1).

### 2.2. Genetic Analyses

The genetic analyses were performed on *A1* SNPs rs11240569, rs708727, and rs823156 in the cohort of 508 PD patients (vs. 150 patients in the pilot study) and the cohort of 472 controls (vs. 120 controls in the pilot study) [37]. Thus, the numbers of the PD patients and of the control probands were increased in this study by 3.4-fold and 3.9-fold, respectively, in comparison with the pilot study. In the sub-cohort of 96 PD patients and 100 controls, we also examined *A1* SNPs rs9438393, rs56152218, and rs61822602 (first identified in the PD sub-cohort by the Sanger sequencing and afterwards by RFLP analysis in the sub-cohort of controls). They were not analyzed in the pilot study [37]. The allele and genotype count and frequencies (fq) for each particular *A1* SNP in the PD and the control cohorts are summarized in Table 3. The minor allele fq was, for rs11240569 (G > A) in our total cohort (PD cases + control probands), roughly comparable with the minor allele total population fq range (MATPFR) reported by the gnomAD and ExAC databases, as follows: fq_o_ (observed) vs. fq_r_ (reported) = 33% vs. 29–30%. The minor allele fq for rs708727 (G > A) in our total cohort was clearly higher than MATPFR in the gnomAD and ExAC databases (fq_o_ vs. fq_r_ = 40% vs. 29–30%) but was comparable with the rs708727 minor allele fq reported for the European population in the ALFA database (41%). The rs823156 (A > G) minor allele fq in our total cohort was 17% and was thus lower than MATPFR in the gnomAD and ExAC databases (23–30%) but was comparable with the rs823156 minor allele fq reported for the European population in the ALFA database (18%). The frequency of the minor allele of rs9438393 (A > G) in the total cohort was 40% and was, therefore, notably higher than the MATPFR of 26–29% reported in the gnomAD and TOPMED databases, but was comparable with the rs9438393 minor allele frequency reported in the ALFA database for the European population (41%). The minor allele of rs56152218 (T > C) in the total cohort was present with an fq of 38%, which is within the MATPFR of 32–46% reported by the ALSPAC, TOPMED, and ALFA (European population) databases. Interestingly, in the Vietnamese, Korean, and Quatari populations, the minor allele is T and not C, as observed in the European population [https://www.ncbi.nlm.nih.gov/snp/?term=rs56152218, accessed on 2 August 2021)]. The fq of the minor allele in rs61822602 (G > T) was found to be 12%. This is comparable with the T allele frequencies reported by the ALSPAC and TWINSUK databases (12% and 13%, respectively) but is far higher than the fq of the T allele reported in the European population in the ALFA database (6%).

All tested *A1* SNPs in our PD and control cohorts were in Hardy–Weinberg equilibrium (HWE; Table 4).

Next, we calculated the odds ratios (OR) of the minor allele and of genotypes containing the minor allele for each tested SNP. These results are summarized in Table 5. The GA genotype in rs708727 was associated with PD (OR = 1.42 (1.08–1.87), *p* = 0.01) in our population. Furthermore, we tested the association of particular genotypic combinations for each tested SNP with PD in recessive, dominant, and completely over-dominant genetic models (Table 6). Coherent with previous data, we identified an association of the rs708727 minor allele (A) with PD in dominant (GG vs. GA + AA) and completely over-dominant (GG + AA vs. GA) genetic models (OR_D_ = 1.36 (1.05–1.77), *p* = 0.02 and OR_COD_ = 1.34 (1.04–1.72), *p* = 0.02, respectively). The remaining SNPs showed no association with PD in the tested genetic models (Table 6).

We also tested the equality of population proportions of any genotypic combination composed of duplets, triplets, quadruplets, quintuplets, or sextuplets of the tested SNPs in the PD cohort (*N* = 96) and the cohort of controls (*N* = 100) with the aim of examining the size of the effect of the interactions among the tested *A1* SNPs toward the susceptibility for developing PD in our population. In all, a total of 12 genotypic combinations (two duplets, seven triplets, and three quadruplets, Table 7) with significantly (*p* < 0.05, 10 genotypes; *p* < 0.06, two genotypes) different counts in the PD and control cohorts were identified (Table 7). Following Cohen’s criteria [39], which describe the differences in proportions, only triplet GG_(rs708727)_ + AG_(rs823156)_ + CC_(rs61822602)_ out of the 12 genotypes showed the “medium” size difference defined by Cohen’s *h_(2arcsin√prp1–2arcsin√prp2)_* ≥ 0.5. The *h* value for the remaining 11 genotypes ranged between 0.32 and 0.46 and was thus within the *h* interval from 0.2 to 0.5, which defines small differences in proportions (Table 7) [39]. Therefore, triplet GG_(rs708727)_ + AG_(rs823156)_ + CC_(rs61822602)_ might be clinically meaningful, and future examinations of this genotype with regard to PD susceptibility should be conducted. For rs11240569, rs708727, and rs823156, we performed the same type of analysis with source data from the cohort of 508 PD patients and the cohort of 472 controls. Four genotypes, two duplets (GG_(rs11240569)_ + AG_(rs708727)_, GG_(rs708727)_ + AG_(rs823156)_) and two triplets (GG_(rs11240569)_ + GG_(rs708727)_ + AA_(rs823156)_, (GG_(rs11240569)_ + GG_(rs708727)_ + AG_(rs823156)_), with significantly (*p* < 0.05) different counts in the PD and control cohorts, were identified. Cohen’s *h* calculated for each of the four genotypes was below the threshold of 0.2 [39], and thus, the difference between the population proportions between the tested groups was, in all four cases, negligible.

**Figure 2:**
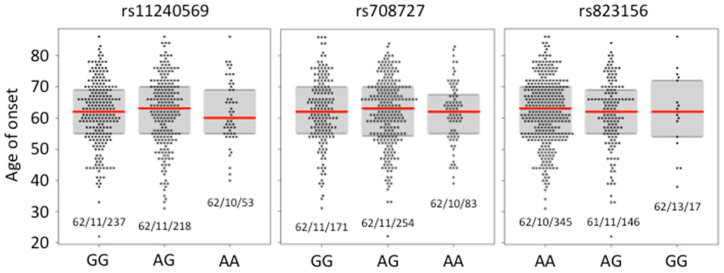
Correlation between the particular genotypes at each tested *A1* SNP with age of onset of PD in cohort of 508 PD patients. Red line indicates median. Numbers below each plot indicate mean/SD/*N*.

**Figure 3:**
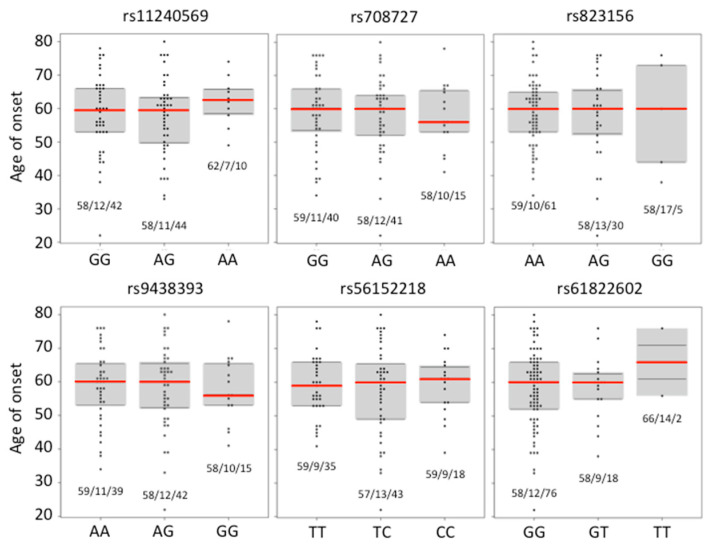
Correlation between the particular genotypes at each tested *A1* SNP with age of onset of PD in sub-cohort of 96 PD patients. Red line indicates median. Numbers below each plot indicate mean/SD/*N*.

Ageing, followed by male gender, are considered to be the most prominent risk factors for the onset of idiopathic PD [37]. In our pilot study, the age of onset of idiopathic PD in the cohort of 150 PD patients was not correlated with the presence of any genotypic combination for SNPs rs11240569, rs708727, and rs823156 [37]. Here, we have correlated the age of onset of PD with (1) the presence of each genotypic combination for SNPs rs11240569, rs708727, and rs823156 in the group of 508 PD patients (Figure 2), and (2) with the presence of each genotypic combination for SNPs rs11240569, rs708727, rs823156, rs9438393, rs56152218, and rs61822602, in the sub-cohort of PD patients, randomly selected from the PD cohort for *A1* promoter sequencing (Figure 3). With regard to the age of onset, a one-way ANOVA analysis revealed that there was no significant (*p* < 0.05) difference between the genotypic sub-populations for each of the tested SNPs in both the large PD cohort and in the sub-cohort of PD patients. Thus, any particular genotype in the tested SNPs obviously does not influence the age of onset of the idiopathic form of PD. This is in full agreement with the conclusion drawn in our pilot study [37].

We have also performed the same type of analysis for each of the tested SNPs in sub-populations of women and men that were derived from the large cohort (*N* = 508) and the sub-cohort (*N* = 96) of PD patients. As demonstrated in supplemental Figures S1–S3, no significant (*p* < 0.05) association between the age of onset and the presence of any particular genotype combination in the tested SNPs rs11240569, rs708727, rs823156, rs9438393, rs56152218, and rs61822602 in the gender-split sub-groups was derived either from the large PD cohort (*N_M_* = 306, *N_F_* = 202) or from the PD sub-cohort selected for *A1* promoter sequencing (*N_M_* = 54, *N_F_* = 42).

### 2.3. RandomForest Machine Learning (RF-ML)

All of the *A1* SNPs were tested for their ability to discriminate between PD patients and controls with RF-ML. The RF-ML algorithm was trained using our data, and the discriminative importance of individual SNPs by a technical construct, known as graph depth, was evaluated [37,40]. As in our pilot study [37], the predictive ability of the tested SNPs was visualized and quantified by ROC (receiver operating characteristic) curves and by AUC (area under ROC curve), respectively. The discriminative ability of predictors is given within the interval of AUC from 100% (maximal discriminative ability) down to 50% (minimal discriminative ability); AUC < 50% corresponds to no discriminative ability.

The RF algorithm was trained in the following modes: (1) with three or six (Table 8) particular *A1* SNPs (each SNP, three genotypes (A^M^A^M^/A^M^A^m^/A^m^A^m^, where A^M^ stands for major allele and A^m^ for minor allele), as predictor (three or six RF models, one for each SNP), (2) with genotypic duplets of the paired SNPs as predictors (three RF models (three SNPs) or 15 RF models (six SNPs), one for each pair of SNPs; Table 8), (3) with genotypic triplets of the three SNPs as predictors (one RF model (three SNPs) or 20 RF models (six SNPs), one for each triplet of SNPs; Table 8), (4) with genotypic quadruplets of the six SNPs as predictors (15 RF models, one for each quadruplet; Table 8), (5) with genotypic quintuplets of the six SNPs as predictors (six RF models, one for each quintuplet; Table 8), and (6) with a genotypic sextuplet (one RF model, Table 8).

Thus, when singletons, duplets, triplets, quadruplets, quintuplets, and the sextuplet of *A1* SNPs were used as predictors, they carried no ability to discriminate between the PD patients and the controls (Table 8). Hence, according to RF-ML analysis, and in agreement with the pilot study [37], the *A1* SNPs have no potential to serve as discriminators between controls and PD patients and, with regard to PD, carry no predictive or diagnostic value in the Slovak population.

## 3. Discussion

The *PARK16* locus has gained attention in the scientific community because of its association with PD and its discussed role in defining susceptibility to this complex ailment. In 2019, we reported a pilot study in which we analyzed the association of the three *A1* SNPs, namely rs11240569, rs708727, and rs823156, with the idiopathic form of PD in the Slovak (Western Slavs) population. The reported association of rs11240569 and rs823156 with susceptibility to PD mainly in Asian/Oriental populations was not found in our study [37]. No association could be confirmed by means of frequentist statistic (conservative genetic analyses) or by RF-ML analysis [37].

The major limitation of our pilot study was, however, the relatively low number of patients/probands in the PD and control cohorts (150 and 120, respectively). We emphasized that, from a statistical point of view, the data had to be interpreted cautiously because of the small sample size in both of the cohorts and because of the low statistical power of the performed analyses [37]. Nevertheless, by utilizing the ML approach, which requires considerably smaller sample sizes than conventional frequentist statistics or approximate Bayesian computation, we were able to examine with confidence the ability of particular *A1* SNPs to discriminate between PD patients and controls. In all instances, the ML approach revealed essentially zero diagnostic and predictive relevance of SNPs rs11240569, rs708727, and rs823156 in the Slovak population [37].

In this study, we have examined not only the three aforementioned SNPs, but also the three SNPs (rs9438393, rs56152218, and rs61822602) localized within the promoter region of *A1*. These SNPs have been identified by the sequencing of 96 PD samples followed by the RFLP analysis of the control samples and the RFLP verification of the sequenced PD samples. Our genetic analyses have revealed essentially no association of any of the three newly studied SNPs with PD. The same is true for rs11240569 and rs823156, which have been analyzed in the PD and control sub-cohorts (96/100) and also in the cohorts of 508 PD patients and 472 controls. These results are in complete agreement with the outcome of the pilot study [37]. However, in the large cohorts, rs708727 has been shown to be associated with an increased risk of PD in the Slovak population.

To our best knowledge, no study has as yet directly associated *A1* SNP rs708727 with an altered risk for developing PD [19,37]. In our enlarged study (when compared with the pilot study [37]), the minor allele A in rs708727 is associated with an increased risk of PD onset when tested in dominant [40] (OR_D_ = 1.36 (1.05–1.77), *p* = 0.02) or completely over-dominant [41] (OR_COD_ = 1.34 (1.04–1.72), *p* = 0.02) models (Table 5). Thus, the presence of the minor allele (A) in rs708727 might be associated with an increased risk of developing PD. Whereas Sanchez-Mut et al. [27] and Wang et al. [42] have clearly shown that the presence of the minor allele A in rs708727 alters the methylation of the *PM20D1* promoter (and, thus, its expression) in a dose-dependent (quantitative) manner, the best-fitting genetic models in our study indicate that the presence of one rs708727 A allele is sufficient to alter the susceptibility to the onset of PD. PD-susceptibility (phenotype), with regard to whether one or two copies of the minor allele are present in rs708727 (genotype), remains to be further elucidated in detail.

Sanchez-Mut et al. have identified *PM20D1* (localized within the *PARK16* locus and encoding for the peptidase M20-domain containing protein one enzyme with both hydrolase and peptidase activities; N-fatty acyl amino acid synthase/hydrolase) as being a methylation and expression quantitative trait locus (mQTL) coupled to an AD-risk associated haplotype, which displays enhancer-like characteristics and contacts the *PM20D1* promoter via a haplotype-dependent, CTCF (CCCTC-binding-) transcription-factor-mediated chromatin loop [27]. By comparing samples from healthy controls and patients with advanced-stage AD, they have found that *PM20D1* consistently displays promoter hypermethylation in patients with AD [27]. *A1* SNP rs708727 correlates with the levels of *PM20D1* DNA methylation in the human frontal cortex and hippocampus [27], as does SNP rs960603 [27]. Wang and colleagues, in their work on peripheral blood, have acquired data confirming that *PM20D1* is an mQTL mediated primarily by the AD-risk associated *A1* SNP rs708727 [42]. Furthermore, their longitudinal data demonstrate that hypomethylation occurs before the symptomatic onset of AD, conceivably to facilitate the increasing expression of *PM20D1* in order to activate its protective function [42]. AD progress is hallmarked by an increasing level of methylation in the CpG islands in the DMR (differentially methylated region) of the *PM20D1* promoter in AD patients, ultimately leading to the inhibition of gene transcription and expression [27,42,43]. PM20D1 has also been associated with diabetes [44], obesity [45], and multiple sclerosis [46], and since it is localized within the *PARK16* locus, its possible involvement/association with PD is assumed [47].

Despite a lack of molecular mechanistic analyses, we speculate, in light of our current data, that AD and PD (and other less frequent neurodegenerative diseases) share not only “well-known” pathophysiological mechanisms (e.g., disturbed mitophagy, retromer, and proteasome functions), but also epigenetic mechanisms, such as *A1* rs708727-dependent regulation of *PM20D1* expression [27,42]. Our work indirectly adds to the need for the detailed elucidation of the role of endogenous *N*-acyl amino acids (NAAs) that are metabolized by PM20D1 in the pathoetiology of PD and other neurodegenerative disorders. NAAs and *N*-acyl conjugates of neurotransmitters (NAANs) are now known to play an important role in neuromodulation [48,49]. Evidence linking *PM20D1* expression with *N*-acyl dopamine (NADA) has been provided by the recent work of Song et al. These authors have shown that the deletion of kir6.2 (a pore-forming subunit of the ATP-sensitive K^+^ channels) leads to a reduced count of mitochondria and lowered ATP production via an increase in the levels of PM20D1 and of agents uncoupling mitochondrial respiration, including NADA, in the murine midbrain [49].

NADA is a potent inhibitor of 5-lipoxygenase (5-LOX) and has a distribution limited to the brain with levels being the highest in the striatum and very low elsewhere [48,50,51]. 5-LOX catalyzes the synthesis of leukotriene or 5-HpETE (5-hydroperoxyeicosatetraenoic acid) from arachidonic acid and has been associated with neurodegeneration (AD and PD) via its involvement in neuroinflammation [51,52]. We therefore suggest that the decreased *PM20D1* expression, the subsequently lower abundance of NADA, and the increased activity of 5-LOX significantly contribute to the pathology of PD (and other neurodegenerative diseases).

Sanchez-Mut and coworkers have demonstrated that the overexpression of *PM20D1* in the murine AD hippocampus results in learning improvement, whereas its knock-down increases the amyloid plaque load [27]. Both the Lewy-type and Alzheimer-type pathologies are important in PD-related dementia [53]. A significant pool of PD patients suffers from worsening dementia during the course of the disease [54]. Taking these observations into consideration we speculate, whether the rs708727-linked activity that silences PM20D1 contributes to the PD-dementia onset, and whether the monitoring of PM20D1 activity can serve as a prognostic parameter for the onset of PD-dementia.

Previously published work has suggested the involvement of Mg^2+^ transporters in the pathoetiology of PD [18,19,20,21,22,55]. A1, being the key player in the Mg homeostasis of somatic cells, has been linked to PD directly [20,21,22]. Point mutations in *A1* leading to substitutions p.A350V, p.R244H, and p.R285Q have been putatively associated with PD, and both the lack of function and the loss of function mutations in *A1* are assumed to have detrimental consequences in neurons, thereby contributing to the PD phenotype [20,21,22]. This work indirectly points toward a possibility that not only perturbations of A1 core function (Na^+^/Mg^2+^ exchange), but also *A1*-linked (rs708727) epigenetic regulation of *PM20D1* expression (and activity), both contribute to pathoetiology of PD.

In this study we have identified genotypic triplet GG_(rs708727)_ + AG_(rs823156)_ + CC_(rs61822602)_ as being potentially clinically meaningful (*h* ≥ 0.5). Interestingly, rs708727 with genotype GG is part of the triplet. However, in light of previous research, the genotype containing the minor allele A in rs708727 would be expected to be linked to a potential PD risk associated with this triplet. Currently we are not able to comment on any molecular/genetic/epigenetic interactions involving the SNPs in the triplet GG_(rs708727)_ + AG_(rs823156)_ + CC_(rs61822602)_, and thus, on any putative contribution of this triplet to a sum of the risk of PD onset.

In our pilot study, we utilized RF-ML to evaluate and interpret our data [37]. The major advantage of RF-ML data analysis is twofold, as follows: (1) it permits the discriminative ability of SNPs between PD patients and controls to be quantified [37], and (2) it requires lower sample sizes for the evaluation of the discriminative importance of individual SNPs or their combinations [37]. Furthermore, RF-ML bypasses the *p*-value problem often associated with larger samples, even when they are available [56]. As in our previous report, none of the tested *A1* SNPs have been shown to have the power to discriminate between PD patients and non-PD probands in our cohort (Table 8). Thus, we can assume that none of the tested *A1* SNPs are suitable for serving as a PD/non-PD discriminator in the Slovak population.

Regarding the association of *A1* rs708727 with altered risk for PD, the outcome of our RF-ML analysis seems to be, on first sight, contradictory (Table 5 and Table 6 vs. Table 8). Jakobsdottir et al. in their logistic regression and ROC curve analyses showed that even strong genetic associations do not automatically guarantee effective discrimination between cases and controls [57]. In spite of being poor classifiers, SNPs with significant OR might be very valuable for establishing etiological hypotheses [57]. In our case, *A1* SNP rs708727 carries no classification power regarding PD, thus, it is of no clinical importance. However, its weak, but significant association with the altered risk for PD allowed us to speculate about involvement of rs708727 in the pathoetiology of PD in a similar or the same way as it is involved in AD [27].

## 4. Materials and Methods

### 4.1. Study Participants (Basic Characteristics)

In total, 980 probands were included in the study (508 PD patients and 472 controls, fulfilling inclusive criteria). The idiopathic form of PD was diagnosed by neurologists in five PD diagnostic centers (in Martin, Bratislava, Trencin, Zvolen, and Kosice) according to the PD diagnostic criteria of the MDS (Movement Disorder Society). All patients were treated with standard anti-PD therapy. The average age of the PD patients was 68.4 ± 9.6 years. The average age of the disease onset was 61.7 ± 10.7 years. The youngest case was diagnosed at the age of 34 and the oldest case at 89 years. The PD group consisted of 202 female (F) and 306 male (M) patients, and thus, the F:M ratio was 1:1.5.

The control cohort of probands consisted of outward and inward patients from the Clinic of Occupational Medicine and Toxicology (University Hospital Martin (UHM)) and the Neurology Clinic (UHM). Only those patients who had not been previously diagnosed with any neurodegenerative and neuropsychiatric disease, such as *diabetes mellitus*, or osteoporosis (all maladies putatively associated with altered *A1* expression and deregulation of A1 function), were enrolled into the control study group. The average age of the control probands was 68.3 ± 11.6 years. The control group consisted of 208 female and 264 male individuals, and thus, the F:M ratio was 1:1.3 in the control group.

The sub-cohort of PD patients, in which the *A1* promoter region was sequenced, consisted of 96 randomly selected subjects. The F:M ratio was 1:1.3 (42 female and 54 male). The average age of patients in the PD sub-cohort was 67.0 ± 9.5 years. The control sub-cohort consisted of 41 females and 59 males, thus the F:M ratio was 1:1.4. The average age of probands in the control sub-cohort was 59.8 ± 5.0 years.

This study was approved by the Ethical Committee at the Jessenius Faculty of Medicine, Comenius University (JFM CU). Approval was recorded under ID: EK 66/2019. All study participants signed informed consent forms.

### 4.2. Sample Processing

Blood samples were collected into EDTA-treated BD Vacutainer^®^ tubes (Becton, Dickinson and Company, Franklin Lakes, NJ USA). Genomic DNA was isolated from fresh (UHM) or frozen blood samples (other PD centers) by using the Wizard^®^ Genomic DNA Purification Kit (Promega Corporation, Maddison, WI, USA) according to the manufacturer’s protocol. Isolated DNA samples were stored at −80 °C.

### 4.3. Genotyping

Genotyping was performed on 358 PD samples and 352 control samples. Results from these experiments were analyzed together with the results previously reported in Cibulka et al. [37]. SNPs rs11240569, rs708727, and rs823156 were analyzed by using TaqMan^®^ genotyping probes C_34251_20/rs11240569, C_375742_10/rs823156, and C_9238453_10/rs708727 (all Thermo Fisher Scientific, Waltham, MA USA) in the same manner as reported previously [37].

### 4.4. Sanger Sequencing

The promoter region was divided into four overlapping fragments/amplicons, as it was too long for a single sequencing run. Before being sequenced, the target regions were amplified. Primers were designed by using the online tool Primer3Plus [https://primer3plus.com/cgi–bin/dev/primer3plus.cgi (accessed on 3 May 2018)]. Each pair of primers was checked for the presence of multiple amplification products by the PCR online tool UCSC [http://www.genome.ucsc.edu/cgi–bin/hgPcr (accessed on 3 May 2018)]. The primers and PCR programs used for amplification of the four fragments are summarized in supplemental Tables S1 and S2. Compositions of master mixes for each fragment are summarized in SA3. Fragments 3 and 4 have a high content of G and C nucleotides (67.4% and 71.9%, respectively). The reaction yield was increased by addition of the 10× G-C Rich Enhancer (Solis Biodyne, Tartu, Estonia). The PCR product was purified by using NucleoSpinTM Gel and a PCR Clean-up kit (Macherey-Nagel GmbH&Co. KG, Düren, Germany). In the next step, the purified PCR product was diluted to an appropriate concentration for pre-sequencing PCR (SA4, SA5). Only forward (fw) primers were used in the pre-sequencing PCR. The reaction mix also included BigDye Terminator v3.1 (Applied Biosystems, Waltham, MA, USA) and dideoxynucleotides. As a result, we obtained a mixture of products of various sizes terminated by fluorescence-marked dideoxynucleotides. The products were subsequently purified by using the SigmaSpin Sequencing Reaction Clean-Up kit (Sigma-Aldrich, St. Louis, MO, USA) according to the manufacturer’s protocol. A volume of 3 µL of purified product was transferred into a 96-well plate together with 12 µL high-grade de-ionized formamide (Applied Biosystems, Waltham, MA, USA). The mixture was denatured for 5 min at 95 °C in a thermocycler. Fragments were separated on the 8-microcapillary device ABI 3500 (Applied Biosystems, Waltham, MA, USA). Sequences were exported and visualized by Chromas software (Technelysium Pty Ltd., South Brisbane, Australia). FASTA files were uploaded to BLAST (Basic Local Alignment Search Tool) and aligned to the reference human genome (version GRCh38.p12).

Prediction of transcription factor binding sites and their alterations was performed by the online tool ConSite [available at: http://consite.genereg.net/ (accessed on 3 September 2020)] [38]. Sequences with the major allele and the minor allele were uploaded, and spectra of the transcription factors (TF) were generated. The analysis was run without pre-setting minimum specificity. Changes in TF-binding profiles based on the presence of variants are summarized in Table 2.

### 4.5. RFLP (Restriction Fragment Length Polymorphism) Analysis

The reaction mix components and PCR conditions of the amplified PCR are summarized in SA6, SA7. The online in silico tool NEBCutter 2.0 [http://nc2.neb.com/NEBcutter2/ (accessed on 14 February 2020)] was used to design restriction of the amplicons. The following restriction enzymes were chosen for RFLP analysis: *Hpy*166II (detection of rs9438393), *NIa*III (detection of rs56152218), and *Bmr*I (detection of rs61822602). All enzymes were purchased from New England Biolabs. For variant rs144056491, we were unable to design an RFLP experiment, as no suitable enzyme was available. Following restriction, we expected the fragments summarized in SA8 to form the yield. After restriction, the products were separated by agarose gel electrophoresis (*NIa*III and *Hpy*166II 1% gel; BmrI 2% gel) and then visualized on a PharosFX instrument (Bio-Rad Laboratories). Genotypes for each SNV were determined.

### 4.6. Data Analysis

Data were explored and analyzed using R [R Core Team (2021); R: a language and environment for statistical computing. R Foundation for Statistical Computing, Vienna, Austria. URL https://www.R-project.org/, ver. 4.0.5 (2021-03-31)]. A genetic association study (GAS) and power analysis were performed using R libraries HardyWeinberg [Jan Graffelman (2015); Exploring Diallelic Genetic Markers: The HardyWeinberg Package. Journal of Statistical Software, 64(3), 1-23. URL https://www.jstatsoft.org/v64/i03/], DescTools [Andri Signorell et al. (2021); DescTools: Tools for descriptive statistics. R package version 0.99.41.], epitools [Tomas J. Aragon (2020); epitools: Epidemiology Tools. R package version 0.5-10.1. URL https://CRAN.R-project.org/package=epitools], pwr [Stephane Champely (2020); pwr: Basic Functions for Power Analysis. R package version 1.3-0. URL https://CRAN.R-project.org/package=pwr] and an in-house developed code. RandomForest predictive modeling was performed with R library randomForestSRC [Ishwaran H. and Kogalur U.B. (2021); Fast Unified RandomForests for Survival, Regression, and Classification (RF-SRC), R package version 2.11.0.]. Data were visualized by R library beeswarm [Aron Eklund (2021); beeswarm: The Bee Swarm Plot, an Alternative to Stripchart. R package version 0.3.1. URL https://CRAN.R-project.org/package=beeswarm]. One-way ANOVA was used to test the null hypothesis of equality of the population mean age of onset for the three genotypes sub-populations for each SNP. Findings with a *p*-value below 0.05 were considered statistically significant.

## 5. Conclusions

In summary, our data suggest a weak, but significant association of *A1* SNP rs708727 with PD in dominant and over-dominant genetic models in a Slovak population. None of the other tested *A1* SNPs (rs11240569, rs823156, rs9438393, rs56152218, and rs61822602) associated with the disease in any of the tested genetic models. RF-ML analyses identified all of the tested *A1* SNPs as being poor classifiers/predictors of PD, thus their use in clinical praxis as diagnostic or prognostic markers remain negligible. However, the association of rs708727 with PD allowed us to speculate that PD-risk associated minor allele (G > A) in rs708727 contributes to the disease onset and progression via the derangement of epigenetic regulation of *PM20D1* expression, the mechanism known to play a role in pathoetiology of AD. This hypothesis should be further examined in order to make a conclusive statement. Furthermore, a possible association between PD-associated dementia and rs708727 should be elucidated.

## Figures and Tables

**Table 1:** *A1* SNPs subjected to analysis in this study.

Gene	SNP	Sequence	Allele	Prot. Level	Reference
*A1*	I	C	G > A	p.Thr113Thr	www.ncbi.nlm.nih.gov/snp/?term=rs11240569 (accessed on 2 August 2021)
	II	C	G > A	p.Asn252Asn	www.ncbi.nlm.nih.gov/snp/?term=rs708727 (accessed on 2 August 2021)
	III	NC/intron	A > G		www.ncbi.nlm.nih.gov/snp/?term=rs823156 (accessed on 2 August 2021)
	IV	NC/P	A > G		www.ncbi.nlm.nih.gov/snp/?term=rs9438393 (accessed on 2 August 2021)
	V	NC/P	T > C		www.ncbi.nlm.nih.gov/snp/?term=rs56152218 (accessed on 2 August 2021)
	VI	NC/P	G > T		www.ncbi.nlm.nih.gov/snp/?term=rs61822602 (accessed on 2 August 2021)

Abbreviations: (I) rs11240569, (II) rs708727, (III) rs823156, (IV) rs9438393, (V) rs56152218, (VI) rs61822602, (*A1*) *SLC41A1*, (C) coding, (NC) non-coding, (P) promoter, (SNP) single nucleotide polymorphism.

**Table 2:** Alterations of transcription-factor-binding domains resulting from presence of respective variants.

SNP	Analyzed CS Sequence	TF Change
**IV**	GGCTCCACAGGGACG**T**/**C**TTACCGGTCTTCCCG	+SP1 −FREAC-4
**V**	GCGCTCCAGGCGCAT**A**/**G**GAGCCGGCTCCCGGTT	+YY1 −Gata2
**VI**	ATCCCGCCCCCTCCC**C**/**A**AGTCCCTGATTGGCT	No change
**rs144056491**	ATGGAGGGGGGGG**GG**TGCCACCCAGTCTGC (G > -,GG,GGG)	No change (p50)

Abbreviations: (IV) rs9438393, (V) rs56152218, (VI) rs61822602, (CS) coding DNA strand, (SNP) single nucleotide polymorphism, (TF) transcription factor.

**Table 3:** Allele and genotype count and frequency in PD and control cohorts for tested *A1* SNPs.

SNP	Cohort	Allele	Count	fq (%)	Genotype	Count	fq (%)
(I)	PD	G	692 (210)	68.1 (70)	GG	237(73)	46.7(48)
		A	324 (90)	31.9 (30)	AG	218(64)	42.9(43)
					AA	53(13)	10.4(9)
	C	G	628 (166)	66.5 (69)	GG	214(57)	45.3(48)
		A	316 (74)	33.5 (31)	AG	200(52)	42.4(43)
					AA	58(11)	12.3(9)
(II)	PD	G	596 (179)	58.7 (60)	GG	171 (54)	33.7 (36)
		A	420 (121)	41.3 (40)	AG	254 (71)	50.0 (47)
					AA	83 (25)	16.3 (17)
	C	G	588 (136)	62.3 (57)	GG	193 (40)	40.9 (33)
		A	356 (104)	37.7 (43)	AG	202 (56)	42.8 (47)
					AA	77 (24)	16.3 (20)
(III)	PD	A	836 (243)	82.3 (81)	AA	345 (100)	67.9 (67)
		G	180 (57)	17.7 (19)	AG	146 (43)	28.7 (29)
					GG	17 (7)	3.4 (5)
	C	A	791 (204)	83.8 (85)	AA	330 (87)	69.9 (73)
		G	153 (36)	16.2 (15)	AG	131 (30)	27.8 (25)
					GG	11 (3)	2.3 (2)
(IV)	PD	A	120	62.5	AA	39	40.6
		G	72	37.5	AG	42	43.8
					GG	15	15.6
	C	A	116	58.0	AA	33	33
		G	84	42.0	AG	50	50
					GG	17	17
(V)	PD	T	113	58.85	TT	35	36.5
		C	79	41.15	TC	43	44.8
					CC	18	18.7
	C	T	131	65.5	TT	41	41
		C	69	34.5	TC	49	49
					CC	10	10
(VI)	PD	G	170	88.54	GG	76	79.2
		T	22	11.46	GT	18	18.7
					TT	2	2.1
	C	G	174	87	GG	76	76
		T	26	13	GT	22	22
					TT	2	2

Abbreviations: (I) rs11240569, (II) rs708727, (III) rs823156, (IV) rs9438393, (V) rs56152218, (VI) rs61822602, (C) control, (fq) frequency, (PD) Parkinson’s disease, (SNP) single nucleotide polymorphism. White background, SNPs analyzed in cohort of 508 PD patients and 472 controls; gray background, SNPs analyzed in sub-cohort of 96 PD patients and 100 controls.

**Table 4:** Genotype distribution of all tested *A1* SNPs in PD and control cohorts conforms to Hardy–Weinberg equilibrium.

SNP	rs11240569 (G > A) Cohort		rs708727 (G > A) Cohort		rs823156 (A > G) Cohort	
	PD *N* (O/E)	C *N* (O/E)	PD *N* (O/E)	C *N* (O/E)	PD *N* (O/E)	C *N* (O/E)
GG (com.)	237/235.66	214/20.89				
AG	218/220.68	200/210.22				
AA (rar.)	53/51.66	58/52.89				
*X* ^2^	0.07	1.12				
*p*-val	0.78	0.29				
GG (com.)			171/174.81	193/183.13		
AG			254/246.38	202/221.74		
AA (rar.)			83/86.81	77/67.13		
*X^2^*			0.49	3.74		
*p*-val			0.49	>0.05		
AA (com.)					345/343.94	330/331.40
AG					146/148.12	131/128.20
GG (rar.)					17/15.94	11/12.40
*X^2^*					0.10	0.22
*p*-val					0.75	0.64
**SNP**	**rs9438393 (G > A)** **Cohort**		**rs56152218 (T > C)** **Cohort**		**rs61822602 (G > T)** **Cohort**	
	PD *N* (O/E)	C *N* (O/E)	PD *N* (O/E)	C *N* (O/E)	PD *N* (O/E)	C *N* (O/E)
AA (com.)	39/37.5	33/33.64				
AG	42/45	50/48.72				
GG (rar.)	15/13.5	17/17.64				
*X* ^2^	0.43	0.07				
*p*-val	0.51	0.79				
TT (com.)			35/33.25	41/42.90		
TC			43/46.50	49/45.20		
CC (rar.)			18/16.25	10/11.90		
*X* ^2^			0.54	0.71		
*p*-val			0.46	0.40		
GG (com.)					76/75.26	76/75.69
GT					18/19.48	22/22.62
TT (rar.)					2/1.26	2/1.69
*X* ^2^					0.55	0.08
*p*-val					0.46	0.78

Abbreviations: (com.) common, (C) control, (E) expected, (*N*) number of individuals, (O) observed, (*p*-val) *p*-value, (PD) Parkinson’s disease, (rar.) rare, (SNP) single nucleotide polymorphism. White background, SNPs analyzed in cohort of 508 PD patients and 472 controls; gray background, SNPs analyzed in sub-cohort of 96 PD patients and 100 controls.

**Table 5:** Odds ratios of minor alleles and genotypes containing minor allele at particular *A1* SNPs.

		95% CI					95% CI			
SNP	MA	OR	ll	uL	*p*-Val	Genotype	OR	ll	uL	*p*-Val
**I**	A	0.93	0.77	1.12	0.47	AA	0.83	0.54	1.25	0.40
**(G > A)**						GA	0.98	0.75	1.28	0.95
**II**	A	1.16	0.97	1.40	0.11	AA	1.22	0.84	1.77	0.34
**(G > A)**						**GA**	**1.42**	**1.08**	**1.87**	**0.01**
**III**	G	1.33	0.82	2.17	0.25	GG	1.48	0.68	3.20	0.34
**(A > G)**						AG	1.07	0.81	1.41	0.67
**IV**	G	0.83	0.55	1.24	0.41	GG	0.75	0.32	1.72	0.53
**(A > G)**						AG	0.71	0.38	1.32	0.35
**V**	C	1.33	0.88	2.00	0.18	CC	2.11	0.86	5.16	0.13
**(T > C)**						TC	1.03	0.56	1.89	1.00
**VI**	T	0.87	0.47	1.59	0.65	TT	1.00	0.14	7.28	1.00
**(G > T)**						GT	0.82	0.41	1.65	0.60

Abbreviations: (I) rs11240569, (II) rs708727, (III) rs823156, (IV) rs9438393, (V) rs56152218, (VI) rs61822602, (CI) confidence interval, (ll) lower limit, (MA) minor allele, (OR) odds ratio, (*p*-val) *p*-value, (SNP) single nucleotide polymorphism, (ul) upper limit. White background, SNPs analyzed in cohort of 508 PD patients and 472 controls; gray background, SNPs analyzed in sub-cohort of 96 PD patients and 100 controls.

**Table 6:** Association of particular *SLC41A1* SNPs with PD according to dominant, recessive, and complete over-dominant genetic models.

		95% CI				
SNP	Genetic Model	OR	ll	uL	*p-*Val	z
**I**	GG vs. GA + AA (D)	0.95	0.74	1.22	0.68	0.41
	GG + GA vs. AA (R)	0.83	0.56	1.24	0.36	0.92
	GG + AA vs. GG (COD)	1.02	0.79	1.32	0.86	0.75
**II**	**GG vs. GA + AA (D)**	**1.36**	**1.05**	**1.77**	**0.02**	**2.34**
	GG + GA vs. AA (R)	1.00	0.71	1.41	0.99	0.01
	**GG + AA vs. GA (COD)**	**1.34**	**1.04**	**1.72**	**0.02**	**2.26**
**III**	AA vs. AG + GG (D)	1.10	0.84	1.44	0.50	0.68
	AA + AG vs. GG (R)	1.45	0.67	3.13	0.34	0.95
	AA + GG vs. AG (COD)	1.05	0.80	1.39	0.73	0.34
**IV**	AA vs. AG + GG (D)	0.73	0.68	2.15	0.26	1.12
	AA + AG vs. GG (R)	0.90	0.42	1.93	0.80	0.26
	AA + GG vs. AG (COD)	0.78	0.44	1.37	0.38	0.88
**V**	TT vs. TC + CC (D)	1.21	0.68	1.15	0.51	0.65
	TT + TC vs. CC (R)	2.08	0.91	4.77	0.08	1.73
	TT + CC vs. TC (COD)	0.84	0.48	1.48	0.56	0.59
**VI**	GG vs. GT + TT (D)	0.83	0.43	1.63	0.60	0.53
	GG + GT vs. TT (R)	1.04	0.14	7.55	0.97	0.04
	GG + TT vs. GT (COD)	0.82	0.41	1.64	0.57	0.56

Abbreviations: (I) rs11240569, (II) rs708727, (III) rs823156, (IV) rs9438393, (V) rs56152218, (VI) rs61822602, (CI) confidence interval, (COR) complete over-dominant, (D) dominant, (ll) lower limit, (OR) odds ratio, (*p*-val) *p*-value, (R) recessive, (SNP) single nucleotide polymorphism, (ul) upper limit. White background, SNPs analyzed in cohort of 508 PD patients and 472 controls; gray background, SNPs analyzed in sub-cohort of 96 PD patients and 100 controls.

**Table 7:** The equality of population proportions of joint genotypes in PD patients and in controls: 12 genotypic combinations with significantly (*p* < 0.05) and near significantly (0.05 < *p* < 0.06) different counts in PD and control cohorts are listed.

SNP													
I	II	III	IV	V	IV	PD/C	*X^2^*	df	*p*-Val	*h*	*N_sss_*	sl	pw
						(*N*/*N*)							
AG		AG				15/6	3.79	1	0.05 *	0.318	78	0.06	0.8
AG				GG		8/1	4.45	1	0.04	0.385	53	0.05	0.8
AG	GG	AG				14/5	4.10	1	0.04	0.333	71	0.05	0.8
AG	GG				CC	15/6	3.79	1	0.05 *	0.318	78	0.06	0.8
AG			TT	GG		8/1	4.45	1	0.04	0.385	53	0.05	0.8
AG				GG	CC	8/1	4.45	1	0.04	0.385	53	0.05	0.8
	**GG**	**AG**			**CC**	**12/1**	**8.69**	**1**	**0.003**	**0.522**	**29**	**0.05**	**0.8**
	GG			GG	CC	18/8	4.03	1	0.05	0.322	76	0.05	0.8
		AG	TT		CC	10/1	6.52	1	0.01	0.457	38	0.05	0.8
AG			TT	GG	CC	8/1	4.45	1	0.04	0.385	53	0.05	0.8
	GG	AG	TT		CC	10/1	6.52	1	0.01	0.457	38	0.05	0.8
	GG		TT	GG	CC	18/8	4.03	1	0.05	0.322	76	0.05	0.8

Proportion power calculation for binomial distribution. Abbreviations: (I) rs11240569, (II) rs708727, (III) rs823156, (IV) rs9438393, (V) rs56152218, (VI) rs61822602, (C) control, (*h*) Cohen’s *h*, (df) degrees of freedom, (*N*) number of individuals, (*N*_sss_) sufficient sample size, (*p*-val) *p*-value, (PD) Parkinson’s disease, (pw) power of test, (sl) significance level, (SNP) single nucleotide polymorphism. * 0.05 < *p*-val < 0.06 was considered near significant. Gray/white background coding is used to separate duplets from triplets and quadruplets.

**Table 8:** *A1* SNPs I (rs11240569), II (rs708727), III (rs823156), IV (rs9438393), V (rs56152218), and VI (rs61822602) used as isolated genotypic singletons (predictors, no color background) or as paired predictors in genotypic duplets (gray), in genotypic triplets (darker gray), in genotypic quadruplets (blue), in genotypic quintuplets (cyclamen), or in a genotypic sextuplet (turquoise).

SNPs I–III (*N =* 980)		SNPs I–VI (*N =* 196)			
Predictor	AUC (%)	Predictor	AUC (%)	Predictor	AUC (%)
I	24.7	I	32.1	II–III–V	38.8
II	35.8	II	34.9	II–III–VI	42.1
III	25.6	III	23.8	II–IV–V	44.3
I–II	45.9	IV	27.2	II–IV–VI	33.4
I–III	28.6	V	17.5	II–V–VI	44.5
II–III	44.7	VI	17.4	III–IV–V	44.7
I–II–III	49.9	I–II	47.9	III–IV–VI	47.8
		I–III	38.1	III–V–VI	45.0
		I–IV	42.2	IV–V–VI	43.9
		I–V	19.5	I–II–III–IV	42.0
		I–VI	35.1	I–II–III–V	43.0
		II–III	28.8	I–II–III–VI	45.8
		II–IV	27.7	I–II–IV–V	44.2
		II–V	46.8	I–II–IV–VI	43.9
		II–VI	31.6	I–II–V–VI	44.3
		III–IV	32.7	I–III–IV–V	43.3
		III–V	16.1	I–II–IV–VI	44.9
		III–VI	20.1	I–III–V–VI	44.8
		IV–V	42.2	I–IV–V–VI	40.8
		IV–VI	27.7	II–III–IV–V	41.2
		V–VI	44.7	II–III–IV–VI	42.2
		I–II–III	43.6	II–III–V–VI	44.6
		I–II–IV	42.8	II–IV–V–VI	44.3
		I–II–V	43.5	III–IV–V–VI	46.8
		I–II–VI	46.8	I–II–III–IV–V	40.9
		I–III–IV	42.4	I–II–III–IV–VI	44.2
		I–III–V	43.6	I–II–III–V–VI	44.7
		I–III–VI	47.4	I–II–IV–V–VI	43.7
		I–IV–V	40.7	I–III–IV–V–VI	46.0
		I–IV–VI	39.2	II–III–IV–V–VI	42.5
		I–V–VI	41.1	I–II–III–IV–V–VI	42.6
		II–III–IV	31.2		

The left panel shows the AUC (area under receiver operation curve) calculated for isolated singletons, duplets, and triplet from the source data collected in the large cohort (508 PD cases and 472 controls); the right panel shows the AUC calculated for duplets, triplets, quadruplets, quintuplets, and the sextuplet from the source data collected from the sub-cohort of 96 PD cases and 100 controls). Abbreviations: (SNP) single nucleotide polymorphism.

## Data Availability

The complete dataset is contained within the article. The nature and extent of the included data allows for further meta-analysis. Any information regarding the study is available on request from the corresponding author.

## Supplementary Materials

The following supporting information can be downloaded at: https://www.mdpi.com/article/10.3390/ijms23031604/s1.

## References

[1] Tatarkova Z., de Baaij J.H.F., Grendar M., Aschenbach J.R., Racay P., Bos C., Sponder G., Hoenderop J.G.J., Röntgen M., Turcanova Koprusakova M. (2020). Dietary Mg^2+^ Intake and the Na^+^/Mg^2+^ Exchanger *SLC41A1* Influence Components of Mitochondrial Energetics in Murine Cardiomyocytes. Int. J. Mol. Sci..

[2] Yamanaka R., Tabata S., Shindo Y., Hotta K., Suzuki K., Soga T., Oka K. (2016). Mitochondrial Mg^2+^ homeostasis decides cellular energy metabolism and vulnerability to stress. Sci. Rep..

[3] Dudev T., Grauffel C., Lim C. (2017). How Native and Alien Metal Cations Bind ATP: Implications for Lithium as a Therapeutic Agent. Sci. Rep..

[4] Kolisek M., Zsurka G., Samaj J., Weghuber J., Schweyen R.J., Schweigel M. (2003). Mrs2p is an essential component of the major electrophoretic Mg^2+^ influx system in mitochondria. EMBO J..

[5] Schindl R., Weghuber J., Romanin C., Schweyen R.J. (2007). Mrs2p forms a high conductance Mg^2+^ selective channel in mitochondria. Biophys. J..

[6] Kolisek M., Sponder G., Pilchova I., Cibulka M., Tatarkova Z., Werner T., Racay P. (2019). Magnesium Extravaganza: A Critical Compendium of Current Research into Cellular Mg^2+^ Transporters Other than TRPM6/7. Rev. Physiol. Biochem. Pharmacol..

[7] Kubota T., Shindo Y., Tokuno K., Komatsu H., Ogawa H., Kudo S., Kitamura Y., Suzuki K., Oka K. (2005). Mitochondria are intracellular magnesium stores: Investigation by simultaneous fluorescent imagings in PC12 cells. Biochim. Biophys. Acta.

[8] Sponder G., Abdulhanan N., Fröhlich N., Mastrototaro L., Aschenbach J.R., Röntgen M., Pilchova I., Cibulka M., Racay P., Kolisek M. (2017). Overexpression of Na^+^/Mg^2+^ exchanger SLC41A1 attenuates pro-survival signaling. Oncotarget.

[9] Yamaguchi H., Wang H.G. (2001). The protein kinase PKB/Akt regulates cell survival and apoptosis by inhibiting Bax conformational change. Oncogene.

[10] Vauzour D., Vafeiadou K., Rice-Evans C., Williams R.J., Spencer J.P. (2007). Activation of pro-survival Akt and ERK1/2 signalling pathways underlie the anti-apoptotic effects of flavanones in cortical neurons. J. Neurochem..

[11] Muddapu V.R., Dharshini S.A.P., Chakravarthy V.S., Gromiha M.M. (2020). Neurodegenerative Diseases—Is Metabolic Deficiency the Root Cause?. Front. Neurosci..

[12] Pathak D., Berthet A., Nakamura K. (2013). Energy failure: Does it contribute to neurodegeneration?. Ann. Neurol..

[13] Dharshini S.A.P., Taguchi Y.H., Gromiha M.M. (2019). Investigating the energy crisis in Alzheimer disease using transcriptome study. Sci. Rep..

[14] Twig G., Shirihai O.S. (2011). The interplay between mitochondrial dynamics and mitophagy. Antioxid. Redox Signal..

[15] Zhao W., Zhang W., Ma H., Yang M. (2020). NIPA2 regulates osteoblast function by modulating mitophagy in type 2 diabetes osteoporosis. Sci. Rep..

[16] Nadler M.J., Hermosura M.C., Inabe K., Perraud A.L., Zhu Q., Stokes A.J., Kurosaki T., Kinet J.P., Penner R., Scharenberg A.M. (2001). LTRPC7 is a Mg.ATP-regulated divalent cation channel required for cell viability. Nature.

[17] Kolisek M., Nestler A., Vormann J., Schweigel-Röntgen M. (2012). Human gene *SLC41A1* encodes for the Na^+^/Mg^2+^ exchanger. Am. J. Physiol. Cell Physiol..

[18] Sturgeon M., Wu P., Cornell R. (2016). SLC41A1 and TRPM7 in magnesium homeostasis and genetic risk for Parkinson’s disease. J. Neurol. Neuromed..

[19] Tucci A., Nalls M.A., Houlden H., Revesz T., Singleton A.B., Wood N.W., Hardy J., Paisán-Ruiz C. (2010). Genetic variability at the *PARK16* locus. Eur. J. Hum. Genet..

[20] Kolisek M., Sponder G., Mastrototaro L., Smorodchenko A., Launay P., Vormann J., Schweigel-Röntgen M. (2013). Substitution p.A350V in Na⁺/Mg²⁺ exchanger SLC41A1, potentially associated with Parkinson’s disease, is a gain-of-function mutation. PLoS ONE.

[21] Lin C.H., Wu Y.R., Chen W.L., Wang H.C., Lee C.M., Lee-Chen G.J., Chen C.M. (2014). Variant R244H in Na^+^/Mg^2+^ exchanger SLC41A1 in Taiwanese Parkinson’s disease is associated with loss of Mg^2+^ efflux function. Parkinsonism Relat. Disord..

[22] Li C., Ou R., Chen Y., Gu X., Wei Q., Cao B., Zhang L., Hou Y., Liu K., Chen X. (2021). Mutation analysis of seven SLC family transporters for early-onset Parkinson’s disease in Chinese population. Neurobiol. Aging.

[23] Lin L., Ke Z., Lv M., Lin R., Wu B., Zheng Z. (2017). Effects of MgSO_4_ and magnesium transporters on 6-hydroxydopamine-induced SH-SY5Y cells. Life Sci..

[24] Pihlstrøm L., Rengmark A., Bjørnarå K.A., Dizdar N., Fardell C., Forsgren L., Holmberg B., Larsen J.P., Linder J., Nissbrandt H. (2015). Fine mapping and resequencing of the *PARK16* locus in Parkinson’s disease. J. Hum. Genet..

[25] Wang L., Cheng L., Li N.N., Yu W.J., Sun X.Y., Peng R. (2015). Genetic analysis of *SLC41A1* in Chinese Parkinson’s disease patients. Am. J. Med. Genet. B Neuropsychiatr. Genet..

[26] Madadi F., Khaniani M.S., Shandiz E.E., Ayromlou H., Najmi S., Emamalizadeh B., Taghavi S., Jamshidi J., Tafakhori A., Shahidi G.A. (2016). Genetic Analysis of the *ZNF512B*, *SLC41A1*, and *ALDH2* Polymorphisms in Parkinson’s Disease in the Iranian Population. Genet. Test. Mol. Biomark..

[27] Sanchez-Mut J.V., Heyn H., Silva B.A., Dixsaut L., Garcia-Esparcia P., Vidal E., Sayols S., Glauser L., Monteagudo-Sánchez A., Perez-Tur J. (2018). *PM20D1* is a quantitative trait locus associated with Alzheimer’s disease. Nat. Med..

[28] Chang X.L., Mao X.Y., Li H.H., Zhang J.H., Li N.N., Burgunder J.M., Peng R., Tan E.K. (2011). Association of GWAS loci with PD in China. Am. J. Med. Genet. B Neuropsychiatr. Genet..

[29] Miyake Y., Tanaka K., Fukushima W., Kiyohara C., Sasaki S., Tsuboi Y., Oeda T., Shimada H., Kawamura N., Sakae N. (2016). *PARK16* polymorphisms, interaction with smoking, and sporadic Parkinson’s disease in Japan. J. Neurol. Sci..

[30] Chung S.J., Jung Y., Hong M., Kim M.J., You S., Kim Y.J., Kim J., Song K. (2013). Alzheimer’s disease and Parkinson’s disease genome-wide association study top hits and risk of Parkinson’s disease in Korean population. Neurobiol. Aging.

[31] Yan Y.P., Mo X.Y., Tian J., Zhao G.H., Yin X.Z., Jin F.Y., Zhang B.R. (2011). An association between the PARK16 locus and Parkinson’s disease in a cohort from eastern China. Parkinsonism Relat. Disord..

[32] Mata I.F., Yearout D., Alvarez V., Coto E., de Mena L., Ribacoba R., Lorenzo-Betancor O., Samaranch L., Pastor P., Cervantes S. (2011). Replication of *MAPT* and *SNCA*, but not *PARK16-18*, as susceptibility genes for Parkinson’s disease. Mov. Disord..

[33] Gopalai A.A., Ahmad-Annuar A., Li H.H., Zhao Y., Lim S.Y., Tan A.H., Lim T.T., Eow G.B., Santhi P., Shanthi V. (2016). *PARK16* is associated with PD in the Malaysian population. Am. J. Med. Genet. B Neuropsychiatr. Genet..

[34] Bai Y., Dong L., Huang X., Zheng S., Qiu P., Lan F. (2017). Associations of rs823128, rs1572931, and rs823156 polymorphisms with reduced Parkinson’s disease risks. Neuroreport.

[35] Kolisek M., Launay P., Beck A., Sponder G., Serafini N., Brenkus M., Froschauer E.M., Martens H., Fleig A., Schweigel M. (2008). SLC41A1 is a novel mammalian Mg^2+^ carrier. J. Biol. Chem..

[36] Mastrototaro L., Tietjen U., Sponder G., Vormann J., Aschenbach J.R., Kolisek M. (2015). Insulin Modulates the Na^+^/Mg^2+^ Exchanger SLC41A1 and Influences Mg^2+^ Efflux from Intracellular Stores in Transgenic HEK293 Cells. J. Nutr..

[37] Cibulka M., Brodnanova M., Grendar M., Grofik M., Kurca E., Pilchova I., Osina O., Tatarkova Z., Dobrota D., Kolisek M. (2019). SNPs rs11240569, rs708727, and rs823156 in *SLC41A1* Do Not Discriminate Between Slovak Patients with Idiopathic Parkinson’s Disease and Healthy Controls: Statistics and Machine-Learning Evidence. Int. J. Mol. Sci..

[38] Sandelin A., Wasserman W.W., Lenhard B. (2004). ConSite: Web-based prediction of regulatory elements using cross-species comparison. Nucleic Acids Res..

[39] Cohen J. (1988). Statistical Power Analysis for the Behavioral Sciences.

[40] Deo R.C. (2015). Machine Learning in Medicine. Circulation.

[41] Miko I. (2008). Genetic dominance: Genotype-phenotype relationships. Nat. Educ..

[42] Wang Q., Chen Y., Readhead B., Chen K., Su Y., Reiman E.M., Dudley J.T. (2020). Longitudinal data in peripheral blood confirm that *PM20D1* is a quantitative trait locus (QTL) for Alzheimer’s disease and implicate its dynamic role in disease progression. Clin. Epigenet..

[43] Popovic M., Fiano V., Fasanelli F., Trevisan M., Grasso C., Assumma M.B., Gillio-Tos A., Polidoro S., De Marco L., Rusconi F. (2019). Differentially methylated DNA regions in early childhood wheezing: An epigenome-wide study using saliva. Pediatr. Allergy Immunol..

[44] Larrick J.W., Larrick J.W., Mendelsohn A.R. (2016). Uncoupling mitochondrial respiration for diabesity. Rejuvenation Res..

[45] Long J.Z., Svensson K.J., Bateman L.A., Lin H., Kamenecka T., Lokurkar I.A., Lou J., Rao R.R., Chang M.R., Jedrychowski M.P. (2016). The secreted enzyme PM20D1 regulates lipidated amino acid uncouplers of mitochondria. Cell.

[46] Maltby V.E., Lea R.A., Sanders K.A., White N., Benton M.C., Scott R.J., Lechner-Scott J. (2017). Differential methylation at MHC in CD4^+^ T cells is associated with multiple sclerosis independently of *HLA-DRB1*. Clin. Epigenet..

[47] Simón-Sánchez J., Schulte C., Bras J.M., Sharma M., Gibbs J.R., Berg D., Paisan-Ruiz C., Lichtner P., Scholz S.W., Hernandez D.G. (2009). Genome-wide association study reveals genetic risk underlying Parkinson’s disease. Nat. Genet..

[48] Connor M., Vaughan C.W., Vandenberg R.J. (2010). N-acyl amino acids and N-acyl neurotransmitter conjugates: Neuromodulators and probes for new drug targets. Brit. J. Pharmacol..

[49] Song N., Fang Y., Zhu H., Liu J., Jiang S., Sun S., Xu R., Ding J., Hu G., Lu M. (2021). Kir6.2 is essential to maintain neurite features by modulating PM20D1-reduced mitochondrial ATP generation. Redox Biol..

[50] Tseng C.F., Iwakami S., Mikajiri A., Shibuya M., Hanaoka F., Ebizuka Y., Padmawinata K., Sankawa U. (1992). Inhibition of in vitro prostaglandin and leukotriene biosyntheses by cinnamoyl-beta-phenethylamine and N-acyldopamine derivatives. Chem. Pharm. Bull..

[51] Kang K.H., Liou H.H., Hour M.J., Liou H.C., Fu W.M. (2013). Protection of dopaminergic neurons by 5-lipoxygenase inhibitor. Neuropharmacology.

[52] Shekhar S., Yadav S.K., Rai N., Kumar R., Yadav Y., Tripathi M., Dey A.B., Dey S. (2018). 5-LOX in Alzheimer’s Disease: Potential Serum Marker and In Vitro Evidences for Rescue of Neurotoxicity by Its Inhibitor YWCS. Mol. Neurobiol..

[53] Compta Y., Parkkinen L., O’Sullivan S.S., Vandrovcova J., Holton J.L., Collins C., Lashley T., Kallis C., Williams D.R., de Silva R. (2011). Lewy- and Alzheimer-type pathologies in Parkinson’s disease dementia: Which is more important?. Brain J. Neurol..

[54] Meireles J., Massano J. (2012). Cognitive impairment and dementia in Parkinson’s disease: Clinical features, diagnosis, and management. Front. Neurol..

[55] Lin M., Lucas H.C., Shmueli G. (2013). Research commentary—Too big to fail: Large samples and the p-value problem. Inf. Syst. Res..

[56] Sun Y., Sukumaran P., Singh B.B. (2020). Magnesium-Induced Cell Survival Is Dependent on TRPM7 Expression and Function. Mol. Neurobiol..

[57] Jakobsdottir J., Gorin M.B., Conley Y.P., Ferrell R.E., Weeks D.E. (2009). Interpretation of genetic association studies: Markers with replicated highly significant odds ratios may be poor classifiers. PLoS Genet..

