# Supplementary material for: Alzheimer’s Disease-Associated SNP rs708727 in SLC41A1 May Increase Risk for Parkinson’s Disease: Report from Enlarged Slovak Study
Source: Int J Mol Sci. 2022 Jan 29;23(3):1604. doi: 10.3390/ijms23031604 (PMC8835868; doi:10.3390/ijms23031604)
Supplement: Supplementary file 1 [file ijms-23-01604-s001.zip › ijms-1562209-supplementary.pdf]

**Supplemental tables**

**Table S1.** List of primers used for amplification of four *SLC41A1* promoter fragments.

| Fragment | Primers fw/rv | | Amplicon size (bp) |
|---|---|---|---|
| 1 | F1pA1fw | $^{5'}$ACCCCACACCCCGTATTCA$^{3'}$ | 470 |
| | F1pA1rv | $^{5'}$GTCAGTGGAACCTGTGACCAA$^{3'}$ | |
| 2 | F2pA1fw | $^{5'}$GAGCCATGTTCGAATGCCC$^{3'}$ | 476 |
| | F2pA1rv | $^{5'}$GCTCACCCCTGACGAATCC$^{3'}$ | |
| 3 | F3pA1fw | $^{5'}$CATGGGTCTGACCTCCTTTC$^{3'}$ | 509 |
| | F3pA1rv | $^{5'}$ACGCAGTAGGAGGAGTGACG$^{3'}$ | |
| 4 | F4pA1fw | $^{5'}$CACACGCCTCTCAAAGTCTG$^{3'}$ | 533 |
| | F4pA1rv | $^{5'}$CATTTCGCTTCTGCGTTACA$^{3'}$ | |

Abbreviations: (bp) base pair(s).

**Table S2.** Programs used for PCR amplification of respective promoter fragments.

| Fragment(s) | Step of PCR | Temperature (°C) | Duration | Number of cycles |
|---|---|---|---|---|
| 1 & 2 | Initial denaturation | 95 | 1' | 1 |
| | Denaturation | 95 | 30'' | ∨ |
| | Annealing | 62 | 45'' | 30 |
| | Polymerisation | 72 | 45'' | ∧ |
| | Final polymerisation | 72 | 10′ | 1 |
| 3 | Initial denaturation | 95 | 5′ | 1 |
| | Denaturation | 95 | 30'' | ∨ |
| | Annealing | 61.5 | 30'' | 35 |
| | Polymerisation | 72 | 1′ | ∧ |
| | Final polymerisation | 72 | 10′ | 1 |
| 4 | Initial denaturation | 95 | 5′ | 1 |
| | Denaturation | 95 | 45'' | ∨ |
| | Annealing | 54 | 30'' | 36 |
| | Polymerisation | 72 | 40'' | ∧ |
| | Final polymerisation | 72 | 5′ | 1 |

**Table S3.** Composition of PCR mixes.

| Fragment | Component | Volume / Weight per 1 reaction |
|---|---|---|
| 1 & 2 | 2x DreamTaq MM | 15 μL |
| | PCR grade $H_2O$ | 10 μL |
| | Forward primer | 2 μL |
| | Reverse primer | 2 μL |
| | Template | 20 – 200 ng |
| 3 & 4 | 2x DreamTaq MM | 12.5 μL |
| | PCR grade $H_2O$ | 5 μL |
| | Forward primer | 2 μL |
| | Reverse primer | 2 μL |
| | GC-enhancer | 2.5 μL |
| | Template | 20 – 200 ng |

**Table S4.** Program of pre-sequencing PCR common for all four fragments.

| Step of PCR | Temperature (°C) | Step duration | Cycles |
|---|---|---|---|
| Initial denaturation | 95 | 2′ | 1 |
| Denaturation | 95 | 15'' | ⋁ |
| Annealing | 60 | 30'' | 35 |
| Polymerisation | 60 | 4′ | ⋀ |
| Final polymerisation | 60 | 7′ | 1 |

**Table S5.** PCR mix for pre-sequencing PCR common for all four fragments.

| Component | Volume per 1 reaction |
|---|---|
| PCR grade $H_2O$ | 6.5 μL |
| BigDye Terminator | 2 μL |
| Forward primer | 1 μL |
| Template - purified PCR product | 0.5 μL |

**Table S6.** Composition of PCR mix for F3-R4 fragment amplification.

| Component | Volume / Weight per 1 reaction |
|---|---|
| 2x DreamTaq MM | 12.5 μL |
| PCR grade $H_2O$ | 7 μL |
| Forward primer F3 | 1 μL |
| Reverse primer R4 | 1 μL |
| Template | 20 – 200 ng |

Abbreviations: (MM) Master mix.

**Table S7.** PCR Program of F3-R4 fragment amplification.

| Step of PCR | Temperature (°C) | Step duration | Cycles |
|---|---|---|---|
| Initial denaturation | 95 | 5′ | 1 |
| Denaturation | 95 | 45'' | ⋁ |
| Annealing | 62 | 45'' | 35 |
| Polymerisation | 72 | 45'' | ⋀ |
| Final polymerisation | 72 | 10′ | 1 |

**Table S8.** Fragments generated by restriction of F3-R4 amplicons.

| SNP | rs9438393 | rs56152218 | rs61822602 |
|---|---|---|---|
| Genotype | | | |
| $A^MA^M$ | 263 + 601 bp | 864 bp | 70 + 218 + 576 bp |
| $A^MA^m$ | 263 + 601 + 864 bp | 393 + 471 + 864 bp | 70 + 218 + 576 + 646 bp |
| $A^mA^m$ | 864 bp | 393 + 471 bp | 218 + 646 bp |

Abbreviations: ($A^M$) major allele, ($A^m$) minor allele, (bp) base pair(s), (SNP) single nucleotide polymorphism.

**Supplemental figures**

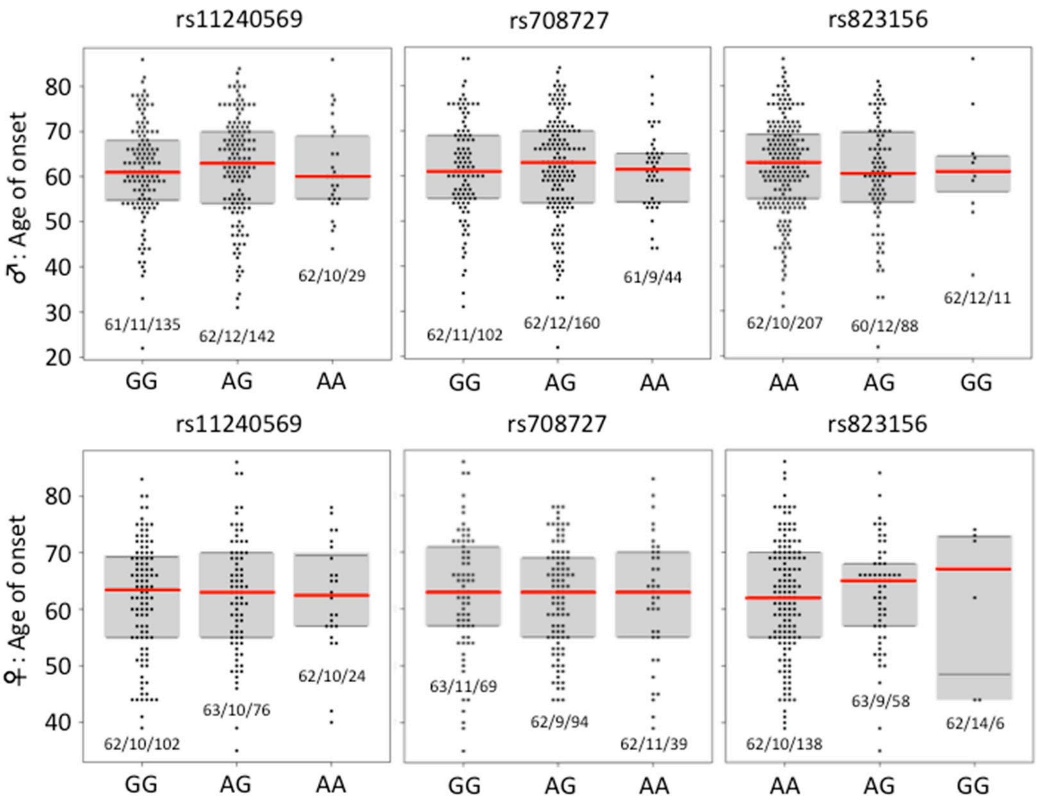


**Figure S1.** Correlation between the particular genotypes at each tested *A1* SNP with age of onset of PD in groups of male ($N$ = 306) and female ($N$ = 202) PD patients. Red line indicates median. Numbers below each plot indicate mean/SD/$N$.

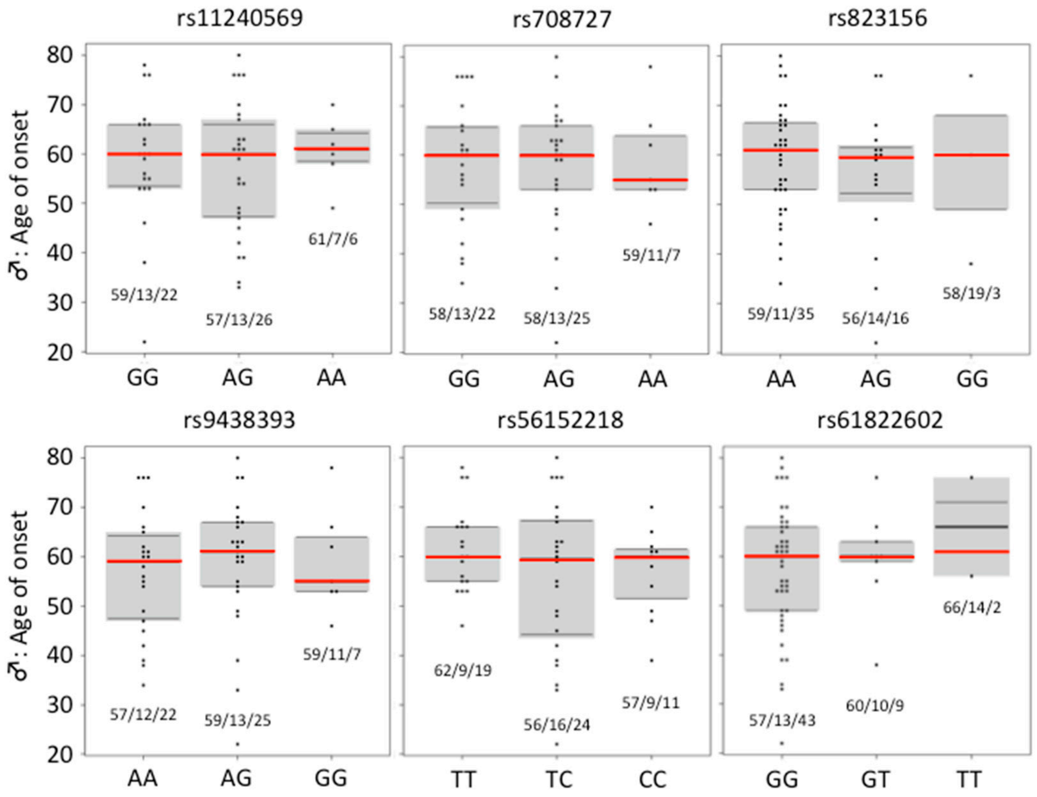


**Figure S2.** Correlation between the particular genotypes at each tested *A1* SNP with age of onset of PD in the group of male PD patients ($N$ = 54). Red line indicates median. Numbers below each plot indicate mean/SD/$N$.

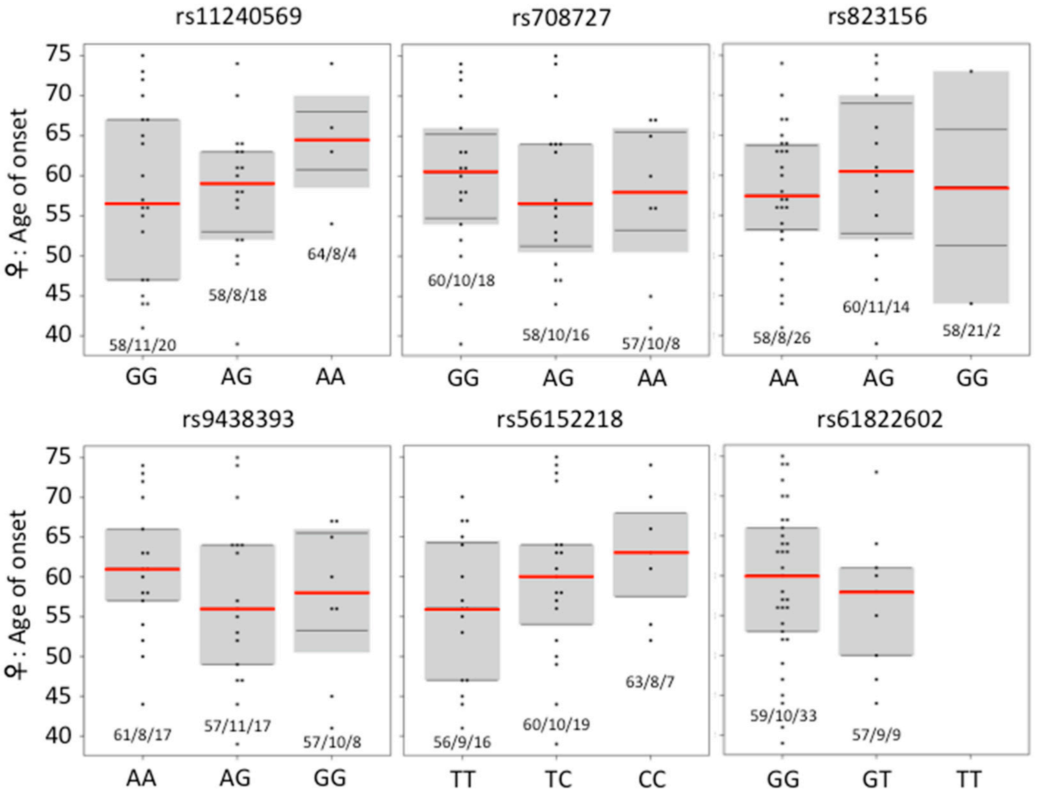


**Figure S3.** Correlation between the particular genotypes at each tested *A1* SNP with age of onset of PD in the group of female PD patients ($N$ = 42). Red line indicates median. Numbers below each plot indicate mean/SD/$N$.
